# Supplementary material for: Women’s education and coverage of skilled birth attendance: An assessment of Sustainable Development Goal 3.1 in the South and Southeast Asian Region
Source: PLoS One. 2020 Apr 21;15(4):e0231489. doi: 10.1371/journal.pone.0231489 (PMC7173780; doi:10.1371/journal.pone.0231489)
Supplement: S1 File — (DOCX) [file pone.0231489.s002.docx]

**Women's education and coverage of skilled birth attendance: An assessment of Sustainable Development Goal 3.1 in the South and Southeast Asian Region**

**Abstract**

**Objective:** The objective of Sustainable Development Goal 3.1 is to reduce the global maternal mortality ratio (MMR) below 70 per 100,000 live births by 2030. One of the indicators for this objective is the proportion of births attended by skilled health attendants (SBA). This study assessed the progress of low- and middle-income countries from South and Southeast Asian (SSEA) region in SBA coverage and evaluated the contribution of women's education in this progression.

**Methods:** The Demographic and Health Surveys were assessed, which included 38 nationally representative surveys on women aged between 15-49 years from 10 selected SSEA region countries in past 30 years. Binary Logistic regression models were fitted adjusting the survey clusters, strata and sampling weights. Meta-analyses were conducted by collapsing effect sizes and confidence intervals of education modeled on SBA coverage.

**Results:** Results indicated that Cambodia, Indonesia and Philippines had over 80% SBA coverage after 2010, whereas Bangladesh and Afghanistan had around 50% coverage. Women with primary, secondary and higher level of education were 1.65, 2.21 and 3.14 times significantly more likely to access SBA care during childbirth respectively as compared to women with no education, suggesting that education is a key factor to address skilled delivery cares in the SSEA region.

**Conclusion:** Evaluation of the existing skilled birth attendance policies at the national level could provide useful insight for the decision makers to improve access to skilled care at birth by investing on women's education in remote and rural areas.

***Keywords:*** Sustainable Development Goal, Maternal mortality ratio, Skilled birth attendants,

Education, Binary Logistic regression, Meta-analysis, Demographic and Health Surveys

**Models**

Each survey was fitted with generalized linear models (GLMs) with binary outcome adjusting for cluster-wise and strata-wise effects (please refer to Tables 1 – 38).

**Afghanistan 2015**

Table 1: Generalized Linear Models (GLMs) with binary outcome fitted to SBA access using sociodemographic factors.

| **Sociodemographic factors** | **OR** | **95% CI** | **p-value** |
| --- | --- | --- | --- |
| **Age** (continuous) | 1.00 | 0.98 - 1.01 | 0.520 |
| **Residence** – (ref: rural)  Urban | 1.48 | 1.04 - 2.11 | 0.030 |
| **Education** (ref: no education)  Primary | 2.23 | 1.73 - 2.88 | <0.001 |
| Secondary | 2.36 | 1.82 - 3.06 | <0.001 |
| Higher | 18.79 | 6.86 - 51.5 | <0.001 |
| **Wealth index** (ref: poorest)  Poorer | 1.89 | 1.63 - 2.19 | <0.001 |
| Middle | 2.45 | 1.87 - 3.20 | <0.001 |
| Richer | 5.06 | 3.98 - 6.42 | <0.001 |
| Richest | 9.66 | 6.33 - 14.75 | <0.001 |
| **Age at first birth** (continuous) | 1.07 | 1.04 - 1.09 | <0.001 |
| **Partner’s education** (ref: no education)  Primary | 1.51 | 1.27 - 1.78 | <0.001 |
| Secondary | 1.79 | 1.51 - 2.13 | <0.001 |
| Higher | 2.04 | 1.56 - 2.66 | <0.001 |
| **Partner’s age** (continuous) | 0.99 | 0.98 - 1.00 | 0.040 |

**Bangladesh 1993**

Table 2: Generalized Linear Models (GLMs) with binary outcome fitted to SBA access using sociodemographic factors.

| **Sociodemographic factors** | **OR** | **95% CI** | **p-value** |
| --- | --- | --- | --- |
| **Age** (continuous) | 0.99 | 0.97 - 1.01 | 0.230 |
| **Residence** – (ref: rural)  Urban | 2.68 | 1.87 - 3.84 | <0.001 |
| **Education** (ref: no education)  Primary | 0.98 | 0.72 - 1.34 | 0.920 |
| Secondary | 2.06 | 1.42 - 2.99 | <0.001 |
| Higher | 6.53 | 3.19 - 13.36 | <0.001 |
| **Wealth index** (ref: poorest)  Poorer | 1.36 | 0.82 - 2.24 | 0.240 |
| Middle | 1.54 | 0.95 - 2.52 | 0.080 |
| Richer | 1.42 | 0.90 - 2.23 | 0.140 |
| Richest | 2.75 | 1.68 - 4.50 | <0.001 |
| **Age at first birth** (continuous) | 1.03 | 0.99 - 1.07 | 0.140 |
| **Partner’s education** (ref: no education)  Primary | 1.26 | 0.94 - 1.70 | 0.120 |
| Secondary | 1.66 | 1.21 - 2.28 | <0.001 |
| Higher | 2.58 | 1.68 - 3.97 | <0.001 |
| **Partner’s age** (continuous) | 0.99 | 0.68 - 1.43 | 0.950 |

**Bangladesh 1996**

Table 3: Generalized Linear Models (GLMs) with binary outcome fitted to SBA access using sociodemographic factors.

| **Sociodemographic factors** | **OR** | **95% CI** | **p-value** |
| --- | --- | --- | --- |
| **Age** (continuous) | 0.99 | 0.96 - 1.01 | 0.230 |
| **Residence** – (ref: rural)  Urban | 2.21 | 1.64 - 2.98 | <0.001 |
| **Education** (ref: no education)  Primary | 1.65 | 1.28 - 2.11 | <0.001 |
| Secondary | 2.44 | 1.80 - 3.30 | <0.001 |
| Higher | 5.45 | 2.81 - 10.56 | <0.001 |
| **Wealth index** (ref: poorest)  Poorer | 1.02 | 0.73 - 1.42 | 0.930 |
| Middle | 1.31 | 0.94 - 1.83 | 0.120 |
| Richer | 1.70 | 1.17 - 2.45 | 0.010 |
| Richest | 2.79 | 1.88 - 4.13 | <0.001 |
| **Age at first birth** (continuous) | 1.08 | 1.05 - 1.12 | <0.001 |
| **Partner’s education** (ref: no education)  Primary | 1.14 | 0.89 - 1.47 | 0.290 |
| Secondary | 1.31 | 0.99 - 1.75 | 0.060 |
| Higher | 2.01 | 1.36 - 2.96 | <0.001 |
| **Partner’s age** (continuous) | 1.00 | 0.98 - 1.02 | 0.730 |

**Bangladesh 1999**

Table 4: Generalized Linear Models (GLMs) with binary outcome fitted to SBA access using sociodemographic factors.

| **Sociodemographic factors** | **OR** | **95% CI** | **p-value** |
| --- | --- | --- | --- |
| **Age** (continuous) | 0.98 | 0.96 – 1.00 | 0.050 |
| **Residence** – (ref: rural)  Urban | 1.86 | 1.49 - 2.30 | <0.001 |
| **Education** (ref: no education)  Primary | 0.99 | 0.78 - 1.24 | 0.910 |
| Secondary | 1.38 | 1.08 - 1.77 | 0.010 |
| Higher | 2.93 | 1.81 - 4.73 | <0.001 |
| **Wealth index** (ref: poorest)  Poorer | 1.05 | 0.80 - 1.38 | 0.720 |
| Middle | 1.05 | 0.76 - 1.44 | 0.760 |
| Richer | 1.24 | 0.93 - 1.66 | 0.150 |
| Richest | 2.51 | 1.85 - 3.41 | <0.001 |
| **Age at first birth** (continuous) | 1.07 | 1.04 - 1.10 | <0.001 |
| **Partner’s education** (ref: no education)  Primary | 1.24 | 1.01 - 1.52 | 0.040 |
| Secondary | 1.54 | 1.24 - 1.93 | <0.001 |
| Higher | 2.18 | 1.58 - 3.00 | <0.001 |
| **Partner’s age** (continuous) | 0.99 | 0.98 - 1.01 | 0.320 |

**Bangladesh 2004**

Table 5: Generalized Linear Models (GLMs) with binary outcome fitted to SBA access using sociodemographic factors.

| **Sociodemographic factors** | **OR** | **95% CI** | **p-value** |
| --- | --- | --- | --- |
| **Age** (continuous) | 0.95 | 0.93 - 0.98 | <0.001 |
| **Residence** – (ref: rural)  Urban | 2.17 | 1.77 - 2.68 | <0.001 |
| **Education** (ref: no education)  Primary | 1.66 | 1.17 - 2.36 | 0.010 |
| Secondary | 2.08 | 1.46 - 2.97 | <0.001 |
| Higher | 4.20 | 2.44 - 7.24 | <0.001 |
| **Wealth index** (ref: poorest)  Poorer | 0.84 | 0.53 - 1.32 | 0.450 |
| Middle | 1.89 | 1.25 - 2.84 | <0.001 |
| Richer | 2.48 | 1.63 - 3.79 | <0.001 |
| Richest | 3.98 | 2.73 - 5.80 | <0.001 |
| **Age at first birth** (continuous) | 1.13 | 1.10 - 1.17 | <0.001 |
| **Partner’s education** (ref: no education)  Primary | 0.98 | 0.73 - 1.32 | 0.880 |
| Secondary | 1.45 | 1.05 - 2.00 | 0.020 |
| Higher | 2.14 | 1.42 - 3.20 | <0.001 |
| **Partner’s age** (continuous) | 1.01 | 0.99 - 1.03 | 0.510 |

**Bangladesh 2007**

Table 6: Generalized Linear Models (GLMs) with binary outcome fitted to SBA access using sociodemographic factors.

| **Sociodemographic factors** | **OR** | **95% CI** | **p-value** |
| --- | --- | --- | --- |
| **Age** (continuous) | 0.95 | 0.93 - 0.97 | <0.001 |
| **Residence** – (ref: rural)  Urban | 1.87 | 1.41 - 2.47 | <0.001 |
| **Education** (ref: no education)  Primary | 1.51 | 1.04 - 2.19 | 0.030 |
| Secondary | 3.07 | 2.10 - 4.48 | <0.001 |
| Higher | 3.53 | 2.27 - 5.48 | <0.001 |
| **Wealth index** (ref: poorest)  Poorer | 1.00 | 0.66 - 1.51 | 0.990 |
| Middle | 1.42 | 0.89 - 2.26 | 0.140 |
| Richer | 2.07 | 1.38 - 3.09 | <0.001 |
| Richest | 3.94 | 2.51 - 6.17 | <0.001 |
| **Age at first birth** (continuous) | 1.15 | 1.11 - 1.19 | <0.001 |
| **Partner’s education** (ref: no education)  Primary | 1.25 | 0.92 - 1.68 | 0.150 |
| Secondary | 1.70 | 1.27 - 2.27 | <0.001 |
| Higher | 3.05 | 2.10 - 4.43 | <0.001 |
| **Partner’s age** (continuous) | 1.02 | 1.00 - 1.04 | 0.070 |

**Bangladesh 2011**

Table 7: Generalized Linear Models (GLMs) with binary outcome fitted to SBA access using sociodemographic factors.

| **Sociodemographic factors** | **OR** | **95% CI** | **p-value** |
| --- | --- | --- | --- |
| **Age** (continuous) | 0.95 | 0.93 - 0.97 | <0.001 |
| **Residence** – (ref: rural)  Urban | 1.84 | 1.53 - 2.22 | <0.001 |
| **Education** (ref: no education)  Primary | 1.29 | 1.00 - 1.66 | 0.050 |
| Secondary | 1.90 | 1.47 - 2.46 | <0.001 |
| Higher | 3.83 | 2.63 - 5.56 | <0.001 |
| **Wealth index** (ref: poorest)  Poorer | 1.18 | 0.92 - 1.51 | 0.190 |
| Middle | 1.55 | 1.21 - 1.98 | <0.001 |
| Richer | 2.34 | 1.84 - 2.99 | <0.001 |
| Richest | 3.50 | 2.62 - 4.68 | <0.001 |
| **Age at first birth** (continuous) | 1.14 | 1.11 - 1.17 | <0.001 |
| **Partner’s education** (ref: no education)  Primary | 1.17 | 0.95 - 1.44 | 0.130 |
| Secondary | 1.48 | 1.20 - 1.83 | <0.001 |
| Higher | 2.15 | 1.64 - 2.82 | <0.001 |
| **Partner’s age** (continuous) | 1.00 | 0.99 - 1.02 | 0.530 |

**Bangladesh 2014**

Table 8: Generalized Linear Models (GLMs) with binary outcome fitted to SBA access using sociodemographic factors.

| **Sociodemographic factors** | **OR** | **95% CI** | **p-value** |
| --- | --- | --- | --- |
| **Age** (continuous) | 0.96 | 0.93 - 1.00 | 0.030 |
| **Residence** – (ref: rural)  Urban | 1.45 | 1.16 - 1.81 | <0.001 |
| **Education** (ref: no education)  Primary | 1.84 | 1.32 - 2.56 | <0.001 |
| Secondary | 2.37 | 1.66 - 3.39 | <0.001 |
| Higher | 3.61 | 2.33 - 5.59 | <0.001 |
| **Wealth index** (ref: poorest)  Poorer | 1.56 | 1.22 - 2.00 | <0.001 |
| Middle | 1.73 | 1.13 - 2.65 | 0.010 |
| Richer | 2.58 | 1.87 - 3.56 | <0.001 |
| Richest | 4.31 | 3.11 - 5.98 | <0.001 |
| **Age at first birth** (continuous) | 1.10 | 1.06 - 1.14 | <0.001 |
| **Partner’s education** (ref: no education)  Primary | 1.13 | 0.90 - 1.42 | 0.290 |
| Secondary | 1.52 | 1.18 - 1.97 | <0.001 |
| Higher | 2.52 | 1.79 - 3.54 | <0.001 |
| **Partner’s age** (continuous) | 1.02 | 1.00 - 1.04 | 0.120 |

**Cambodia 2000**

Table 9: Generalized Linear Models (GLMs) with binary outcome fitted to SBA access using sociodemographic factors.

| **Sociodemographic factors** | **OR** | **95% CI** | **p-value** |
| --- | --- | --- | --- |
| **Age** (continuous) | 0.96 | 0.94 - 0.98 | <0.001 |
| **Residence** – (ref: rural)  Urban | 1.74 | 1.27 - 2.39 | <0.001 |
| **Education** (ref: no education)  Primary | 1.41 | 1.15 - 1.74 | <0.001 |
| Secondary | 2.31 | 1.71 - 3.12 | <0.001 |
| Higher | 2.40 | 0.5 - 11.38 | 0.270 |
| **Wealth index** (ref: poorest)  Poorer | 1.68 | 1.30 - 2.18 | <0.001 |
| Middle | 1.92 | 1.48 - 2.48 | <0.001 |
| Richer | 3.20 | 2.45 - 4.18 | <0.001 |
| Richest | 9.74 | 6.97 - 13.61 | <0.001 |
| **Age at first birth** (continuous) | 1.08 | 1.06 - 1.11 | <0.001 |
| **Partner’s education** (ref: no education)  Primary | 1.39 | 1.08 - 1.79 | 0.010 |
| Secondary | 1.90 | 1.45 - 2.48 | <0.001 |
| Higher | 7.68 | 3.04 - 19.4 | <0.001 |
| **Partner’s age** (continuous) | 1.03 | 1.01 - 1.04 | <0.001 |

**Cambodia 2005**

Table 10: Generalized Linear Models (GLMs) with binary outcome fitted to SBA access using sociodemographic factors.

| **Sociodemographic factors** | **OR** | **95% CI** | **p-value** |
| --- | --- | --- | --- |
| **Age** (continuous) | 0.96 | 0.94 - 0.98 | <0.001 |
| **Residence** – (ref: rural)  Urban | 1.46 | 1.15 - 1.84 | <0.001 |
| **Education** (ref: no education)  Primary | 1.87 | 1.55 - 2.25 | <0.001 |
| Secondary | 3.99 | 3.15 - 5.05 | <0.001 |
| Higher | 2.64 | 0.59 - 11.74 | 0.200 |
| **Wealth index** (ref: poorest)  Poorer | 1.38 | 1.12 - 1.71 | <0.001 |
| Middle | 2.09 | 1.63 - 2.68 | <0.001 |
| Richer | 4.55 | 3.45 - 6.00 | <0.001 |
| Richest | 17.96 | 12.18 - 26.49 | <0.001 |
| **Age at first birth** (continuous) | 1.09 | 1.07 - 1.12 | <0.001 |
| **Partner’s education** (ref: no education)  Primary | 1.13 | 0.90 - 1.41 | 0.290 |
| Secondary | 1.37 | 1.04 - 1.81 | 0.030 |
| Higher | 2.66 | 1.21 - 5.87 | 0.020 |
| **Partner’s age** (continuous) | 1.01 | 0.99 - 1.03 | 0.200 |

**Cambodia 2010**

Table 11: Generalized Linear Models (GLMs) with binary outcome fitted to SBA access using sociodemographic factors.

| **Sociodemographic factors** | **OR** | **95% CI** | **p-value** |
| --- | --- | --- | --- |
| **Age** (continuous) | 0.94 | 0.92 - 0.96 | <0.001 |
| **Residence** – (ref: rural)  Urban | 2.67 | 1.78 - 4.02 | <0.001 |
| **Education** (ref: no education)  Primary | 1.86 | 1.51 - 2.28 | <0.001 |
| Secondary | 2.81 | 1.98 - 3.98 | <0.001 |
| Higher | 6.74e5 | 2.97e5- 1.53 e5 | <0.001 |
| **Wealth index** (ref: poorest)  Poorer | 1.43 | 1.18 - 1.73 | <0.001 |
| Middle | 1.84 | 1.41 - 2.42 | <0.001 |
| Richer | 3.09 | 2.28 - 4.19 | <0.001 |
| Richest | 7.65 | 4.20 - 13.96 | <0.001 |
| **Age at first birth** (continuous) | 1.09 | 1.07 - 1.12 | <0.001 |
| **Partner’s education** (ref: no education)  Primary | 1.22 | 0.98 - 1.53 | 0.080 |
| Secondary | 2.13 | 1.6 - 2.83 | <0.001 |
| Higher | 3.36 | 0.77 - 14.67 | 0.110 |
| **Partner’s age** (continuous) | 1.01 | 1 - 1.03 | 0.160 |

**Cambodia 2014**

Table 12: Generalized Linear Models (GLMs) with binary outcome fitted to SBA access using sociodemographic factors.

| **Sociodemographic factors** | **OR** | **95% CI** | **p-value** |
| --- | --- | --- | --- |
| **Age** (continuous) | 0.94 | 0.91 - 0.97 | <0.001 |
| **Residence** – (ref: rural)  Urban | 1.36 | 0.73 - 2.53 | 0.340 |
| **Education** (ref: no education)  Primary | 1.71 | 1.25 - 2.35 | <0.001 |
| Secondary | 2.62 | 1.68 - 4.09 | <0.001 |
| Higher | 4.68e5 | 1.77 e5 – 1.24 e5 | <0.001 |
| **Wealth index** (ref: poorest)  Poorer | 1.69 | 1.22 - 2.33 | <0.001 |
| Middle | 2.61 | 1.73 - 3.95 | <0.001 |
| Richer | 4.39 | 2.54 - 7.58 | <0.001 |
| Richest | 9.04 | 3.99 - 20.5 | <0.001 |
| **Age at first birth** (continuous) | 1.12 | 1.08 - 1.16 | <0.001 |
| **Partner’s education** (ref: no education)  Primary | 1.20 | 0.85 - 1.69 | 0.290 |
| Secondary | 2.52 | 1.61 - 3.93 | <0.001 |
| Higher | 4.58 | 0.67 - 31.42 | 0.120 |
| **Partner’s age** (continuous) | 1.00 | 0.97 - 1.02 | 0.910 |

**India 1992**

Table 13: Generalized Linear Models (GLMs) with binary outcome fitted to SBA access using sociodemographic factors.

| **Sociodemographic factors** | **OR** | **95% CI** | **p-value** |
| --- | --- | --- | --- |
| **Age** (continuous) | 0.95 | 0.95 - 0.96 | <0.001 |
| **Residence** – (ref: rural)  Urban | 2.03 | 1.78 - 2.32 | <0.001 |
| **Education** (ref: no education)  Primary | 2.04 | 1.87 - 2.24 | <0.001 |
| Secondary | 3.44 | 3.09 - 3.84 | <0.001 |
| Higher | 12.26 | 8.25 - 18.2 | <0.001 |
| **Wealth index** (ref: poorest)  Poorer | 1.25 | 1.12 - 1.39 | <0.001 |
| Middle | 1.87 | 1.67 - 2.10 | <0.001 |
| Richer | 2.89 | 2.54 - 3.28 | <0.001 |
| Richest | 5.19 | 4.37 - 6.16 | <0.001 |
| **Age at first birth** (continuous) | 1.08 | 1.07 - 1.10 | <0.001 |
| **Partner’s education** (ref: no education)  Primary | 1.11 | 1.02 - 1.21 | 0.010 |
| Secondary | 0.92 | 0.84 - 1.01 | 0.070 |
| Higher | 0.91 | 0.77 - 1.06 | 0.220 |
| **Partner’s age** (continuous) | 0.95 | 0.87 - 1.03 | 0.220 |

**India 1998**

Table 14: Generalized Linear Models (GLMs) with binary outcome fitted to SBA access using sociodemographic factors.

| **Sociodemographic factors** | **OR** | **95% CI** | **p-value** |
| --- | --- | --- | --- |
| **Age** (continuous) | 0.92 | 0.91 - 0.94 | <0.001 |
| **Residence** – (ref: rural)  Urban | 2.25 | 1.98 - 2.54 | <0.001 |
| **Education** (ref: no education)  Primary | 1.91 | 1.71 - 2.12 | <0.001 |
| Secondary | 3.01 | 2.73 - 3.31 | <0.001 |
| Higher | 5.25 | 4.28 - 6.44 | <0.001 |
| **Wealth index** (ref: poorest)  Poorer | 1.40 | 1.27 - 1.54 | <0.001 |
| Middle | 2.16 | 1.91 - 2.44 | <0.001 |
| Richer | 3.11 | 2.75 - 3.51 | <0.001 |
| Richest | 5.43 | 4.55 - 6.48 | <0.001 |
| **Age at first birth** (continuous) | 1.14 | 1.12 - 1.16 | <0.001 |
| **Partner’s education** (ref: no education)  Primary | 1.14 | 1.03 - 1.27 | 0.010 |
| Secondary | 1.05 | 0.94 - 1.16 | 0.370 |
| Higher | 1.09 | 0.95 - 1.23 | 0.210 |
| **Partner’s age** (continuous) | 1.02 | 1.01 - 1.02 | <0.001 |

**India 2006**

Table 15: Generalized Linear Models (GLMs) with binary outcome fitted to SBA access using sociodemographic factors.

| **Sociodemographic factors** | **OR** | **95% CI** | **p-value** |
| --- | --- | --- | --- |
| **Age** (continuous) | 0.92 | 0.91 - 0.93 | <0.001 |
| **Residence** – (ref: rural)  Urban | 1.89 | 1.68 - 2.14 | <0.001 |
| **Education** (ref: no education)  Primary | 1.54 | 1.40 - 1.69 | <0.001 |
| Secondary | 2.51 | 2.28 - 2.77 | <0.001 |
| Higher | 9.30 | 6.36 - 13.62 | <0.001 |
| **Wealth index** (ref: poorest)  Poorer | 1.45 | 1.30 - 1.61 | <0.001 |
| Middle | 2.29 | 2.03 - 2.57 | <0.001 |
| Richer | 3.32 | 2.90 - 3.79 | <0.001 |
| Richest | 7.17 | 5.95 - 8.62 | <0.001 |
| **Age at first birth** (continuous) | 1.13 | 1.11 - 1.14 | <0.001 |
| **Partner’s education** (ref: no education)  Primary | 1.18 | 1.07 - 1.31 | <0.001 |
| Secondary | 1.23 | 1.13 - 1.36 | <0.001 |
| Higher | 1.20 | 1.02 - 1.42 | 0.030 |
| **Partner’s age** (continuous) | 1.02 | 1.01 - 1.03 | <0.001 |

**India 2015**

Table 16: Generalized Linear Models (GLMs) with binary outcome fitted to SBA access using sociodemographic factors.

| **Sociodemographic factors** | **OR** | **95% CI** | **p-value** |
| --- | --- | --- | --- |
| **Age** (continuous) | 0.92 | 0.91 - 0.94 | <0.001 |
| **Residence** – (ref: rural)  Urban | 1.08 | 0.91 - 1.29 | 0.380 |
| **Education** (ref: no education)  Primary | 1.27 | 1.12 - 1.45 | <0.001 |
| Secondary | 1.73 | 1.52 - 1.97 | <0.001 |
| Higher | 2.98 | 2.16 - 4.11 | <0.001 |
| **Wealth index** (ref: poorest)  Poorer | 1.53 | 1.35 - 1.73 | <0.001 |
| Middle | 2.32 | 1.99 - 2.70 | <0.001 |
| Richer | 2.99 | 2.40 - 3.73 | <0.001 |
| Richest | 4.95 | 3.71 - 6.62 | <0.001 |
| **Age at first birth** (continuous) | 1.09 | 1.07 - 1.10 | <0.001 |
| **Partner’s education** (ref: no education)  Primary | 1.09 | 0.95 - 1.25 | 0.240 |
| Secondary | 1.33 | 1.18 - 1.50 | <0.001 |
| Higher | 1.43 | 1.14 - 1.79 | <0.001 |
| **Partner’s age** (continuous) | 1.02 | 1.00 - 1.03 | 0.010 |

**Indonesia 1997**

Table 17: Generalized Linear Models (GLMs) with binary outcome fitted to SBA access using sociodemographic factors.

| **Sociodemographic factors** | **OR** | **95% CI** | **p-value** |
| --- | --- | --- | --- |
| **Age** (continuous) | 1.00 | 0.99 - 1.01 | 0.540 |
| **Residence** – (ref: rural)  Urban | 2.33 | 1.72 - 3.17 | <0.001 |
| **Education** (ref: no education)  Primary | 1.61 | 1.24 - 2.09 | <0.001 |
| Secondary | 3.79 | 2.81 - 5.11 | <0.001 |
| Higher | 7.96 | 3.26 - 19.45 | <0.001 |
| **Wealth index** (ref: poorest)  Poorer | 1.74 | 1.45 - 2.08 | <0.001 |
| Middle | 2.37 | 1.93 - 2.91 | <0.001 |
| Richer | 3.14 | 2.48 - 3.98 | <0.001 |
| Richest | 6.85 | 5.10 - 9.19 | <0.001 |
| **Age at first birth** (continuous) | 1.09 | 1.07 - 1.11 | <0.001 |
| **Partner’s education** (ref: no education)  Primary | 1.80 | 1.23 - 2.63 | <0.001 |
| Secondary | 3.20 | 2.19 - 4.68 | <0.001 |
| Higher | 5.40 | 2.99 - 9.77 | <0.001 |
| **Partner’s age** (continuous) | 0.86 | 0.70 - 1.06 | 0.150 |

**Indonesia 2002**

Table 18: Generalized Linear Models (GLMs) with binary outcome fitted to SBA access using sociodemographic factors.

| **Sociodemographic factors** | **OR** | **95% CI** | **p-value** |
| --- | --- | --- | --- |
| **Age** (continuous) | 0.98 | 0.96 – 1.00 | 0.050 |
| **Residence** – (ref: rural)  Urban | 1.09 | 0.81 - 1.48 | 0.570 |
| **Education** (ref: no education)  Primary | 1.35 | 1.00 - 1.83 | 0.050 |
| Secondary | 2.89 | 2.06 - 4.05 | <0.001 |
| Higher | 5.85 | 2.66 - 12.86 | <0.001 |
| **Wealth index** (ref: poorest)  Poorer | 1.79 | 1.40 - 2.30 | <0.001 |
| Middle | 2.37 | 1.75 - 3.21 | <0.001 |
| Richer | 3.60 | 2.51 - 5.18 | <0.001 |
| Richest | 6.51 | 4.21 - 10.05 | <0.001 |
| **Age at first birth** (continuous) | 1.08 | 1.06 - 1.11 | <0.001 |
| **Partner’s education** (ref: no education)  Primary | 2.08 | 1.42 - 3.04 | <0.001 |
| Secondary | 3.95 | 2.64 - 5.92 | <0.001 |
| Higher | 12.50 | 6.12 - 25.53 | <0.001 |
| **Partner’s age** (continuous) | 1.01 | 0.99 - 1.03 | 0.200 |

**Indonesia 2007**

Table 19: Generalized Linear Models (GLMs) with binary outcome fitted to SBA access using sociodemographic factors.

| **Sociodemographic factors** | **OR** | **95% CI** | **p-value** |
| --- | --- | --- | --- |
| **Age** (continuous) | 0.98 | 0.97 – 1.00 | 0.080 |
| **Residence** – (ref: rural)  Urban | 1.50 | 1.14 - 1.97 | <0.001 |
| **Education** (ref: no education)  Primary | 1.75 | 1.19 - 2.56 | <0.001 |
| Secondary | 4.06 | 2.67 - 6.17 | <0.001 |
| Higher | 14.18 | 6.97 - 28.84 | <0.001 |
| **Wealth index** (ref: poorest)  Poorer | 1.96 | 1.63 - 2.37 | <0.001 |
| Middle | 2.83 | 2.26 - 3.55 | <0.001 |
| Richer | 3.76 | 2.89 - 4.88 | <0.001 |
| Richest | 6.84 | 4.59 - 10.19 | <0.001 |
| **Age at first birth** (continuous) | 1.09 | 1.06 - 1.11 | <0.001 |
| **Partner’s education** (ref: no education)  Primary | 1.36 | 0.93 - 1.99 | 0.120 |
| Secondary | 1.98 | 1.32 - 2.99 | <0.001 |
| Higher | 2.64 | 1.42 - 4.88 | <0.001 |
| **Partner’s age** (continuous) | 1.02 | 1.00 - 1.03 | 0.020 |

**Indonesia 2012**

Table 20: Generalized Linear Models (GLMs) with binary outcome fitted to SBA access using sociodemographic factors.

| **Sociodemographic factors** | **OR** | **95% CI** | **p-value** |
| --- | --- | --- | --- |
| **Age** (continuous) | 0.98 | 0.98 - 1.02 | 0.920 |
| **Residence** – (ref: rural)  Urban | 1.17 | 1.17 – 2.00 | <0.001 |
| **Education** (ref: no education)  Primary | 1.86 | 1.86 - 3.99 | <0.001 |
| Secondary | 3.57 | 3.57 - 7.95 | <0.001 |
| Higher | 5.39 | 5.39 - 15.92 | <0.001 |
| **Wealth index** (ref: poorest)  Poorer | 1.97 | 1.97 - 2.93 | <0.001 |
| Middle | 2.78 | 2.78 - 4.62 | <0.001 |
| Richer | 3.61 | 3.61 - 7.27 | <0.001 |
| Richest | 3.46 | 3.46 - 9.21 | <0.001 |
| **Age at first birth** (continuous) | 1.07 | 1.07 - 1.12 | <0.001 |
| **Partner’s education** (ref: no education)  Primary | 0.98 | 0.98 - 2.50 | 0.060 |
| Secondary | 1.59 | 1.59 - 4.24 | <0.001 |
| Higher | 1.69 | 1.69 - 5.74 | <0.001 |
| **Partner’s age** (continuous) | 0.98 | 0.98 - 1.02 | 0.990 |

**Myanmar 2016**

Table 21: Generalized Linear Models (GLMs) with binary outcome fitted to SBA access using sociodemographic factors.

| **Sociodemographic factors** | **OR** | **95% CI** | **p-value** |
| --- | --- | --- | --- |
| **Age** (continuous) | 0.99 | 0.96 - 1.01 | 0.340 |
| **Residence** – (ref: rural)  Urban | 1.89 | 1.11 - 3.22 | 0.020 |
| **Education** (ref: no education)  Primary | 2.32 | 1.76 - 3.08 | <0.001 |
| Secondary | 3.77 | 2.60 - 5.44 | <0.001 |
| Higher | 5.05 | 1.93 - 13.22 | <0.001 |
| **Wealth index** (ref: poorest)  Poorer | 1.47 | 1.15 - 1.87 | <0.001 |
| Middle | 1.84 | 1.29 - 2.61 | <0.001 |
| Richer | 3.31 | 2.19 - 4.99 | <0.001 |
| Richest | 20.80 | 10.74 - 40.25 | <0.001 |
| **Age at first birth** (continuous) | 1.11 | 1.08 - 1.14 | <0.001 |
| **Partner’s education** (ref: no education)  Primary | 1.34 | 1.03 - 1.74 | 0.030 |
| Secondary | 1.81 | 1.33 - 2.45 | <0.001 |
| Higher | 2.07 | 0.78 - 5.51 | 0.140 |
| **Partner’s age** (continuous) | 1.00 | 0.98 - 1.02 | 0.900 |

**Nepal 1996**

Table 22: Generalized Linear Models (GLMs) with binary outcome fitted to SBA access using sociodemographic factors.

| **Sociodemographic factors** | **OR** | **95% CI** | **p-value** |
| --- | --- | --- | --- |
| **Age** (continuous) | 0.93 | 0.89 - 0.97 | <0.001 |
| **Residence** – (ref: rural)  Urban | 4.60 | 2.84 - 7.45 | <0.001 |
| **Education** (ref: no education)  Primary | 1.92 | 1.25 - 2.95 | <0.001 |
| Secondary | 3.02 | 1.97 - 4.62 | <0.001 |
| Higher | 8.72 | 3.36 - 22.6 | <0.001 |
| **Wealth index** (ref: poorest)  Poorer | 1.50 | 0.84 - 2.66 | 0.170 |
| Middle | 1.78 | 0.95 - 3.33 | 0.070 |
| Richer | 1.98 | 1.15 - 3.42 | 0.020 |
| Richest | 4.12 | 2.34 - 7.26 | <0.001 |
| **Age at first birth** (continuous) | 1.15 | 1.09 - 1.21 | <0.001 |
| **Partner’s education** (ref: no education)  Primary | 1.28 | 0.8 - 2.06 | 0.310 |
| Secondary | 2.13 | 1.37 - 3.29 | <0.001 |
| Higher | 2.36 | 1.32 - 4.22 | <0.001 |
| **Partner’s age** (continuous) | 1.03 | 1.00 - 1.06 | 0.090 |

**Nepal 2001**

Table 23: Generalized Linear Models (GLMs) with binary outcome fitted to SBA access using sociodemographic factors.

| **Sociodemographic factors** | **OR** | **95% CI** | **p-value** |
| --- | --- | --- | --- |
| **Age** (continuous) | 0.91 | 0.88 - 0.94 | <0.001 |
| **Residence** – (ref: rural)  Urban | 2.96 | 2.19 - 4.01 | <0.001 |
| **Education** (ref: no education)  Primary | 1.72 | 1.27 - 2.32 | <0.001 |
| Secondary | 3.20 | 2.44 - 4.21 | <0.001 |
| Higher | 6.61 | 2.85 - 15.35 | <0.001 |
| **Wealth index** (ref: poorest)  Poorer | 1.48 | 0.95 - 2.30 | 0.090 |
| Middle | 2.16 | 1.47 - 3.18 | <0.001 |
| Richer | 2.87 | 2.03 - 4.07 | <0.001 |
| Richest | 5.59 | 3.89 - 8.02 | <0.001 |
| **Age at first birth** (continuous) | 1.11 | 1.06 - 1.15 | <0.001 |
| **Partner’s education** (ref: no education)  Primary | 1.21 | 0.93 - 1.58 | 0.170 |
| Secondary | 1.48 | 1.07 - 2.04 | 0.020 |
| Higher | 2.12 | 1.27 - 3.54 | <0.001 |
| **Partner’s age** (continuous) | 1.04 | 1.01 - 1.06 | <0.001 |

**Nepal 2006**

Table 24: Generalized Linear Models (GLMs) with binary outcome fitted to SBA access using sociodemographic factors.

| **Sociodemographic factors** | **OR** | **95% CI** | **p-value** |
| --- | --- | --- | --- |
| **Age** (continuous) | 0.93 | 0.90 - 0.96 | <0.001 |
| **Residence** – (ref: rural)  Urban | 1.70 | 1.24 - 2.33 | <0.001 |
| **Education** (ref: no education)  Primary | 1.59 | 1.17 - 2.14 | <0.001 |
| Secondary | 2.64 | 1.95 - 3.58 | <0.001 |
| Higher | 42.67 | 14.41 - 126.36 | <0.001 |
| **Wealth index** (ref: poorest)  Poorer | 1.79 | 1.17 - 2.75 | 0.010 |
| Middle | 1.57 | 1.05 - 2.35 | 0.030 |
| Richer | 2.83 | 1.93 - 4.14 | <0.001 |
| Richest | 6.44 | 4.10 - 10.13 | <0.001 |
| **Age at first birth** (continuous) | 1.14 | 1.10 - 1.19 | <0.001 |
| **Partner’s education** (ref: no education)  Primary | 1.18 | 0.85 - 1.64 | 0.330 |
| Secondary | 1.17 | 0.89 - 1.53 | 0.260 |
| Higher | 1.46 | 0.90 - 2.35 | 0.130 |
| **Partner’s age** (continuous) | 1.01 | 0.99 - 1.04 | 0.260 |

**Nepal 2011**

Table 25: Generalized Linear Models (GLMs) with binary outcome fitted to SBA access using sociodemographic factors.

| **Sociodemographic factors** | **OR** | **95% CI** | **p-value** |
| --- | --- | --- | --- |
| **Age** (continuous) | 0.91 | 0.89 - 0.94 | <0.001 |
| **Residence** – (ref: rural)  Urban | 1.89 | 1.43 - 2.49 | <0.001 |
| **Education** (ref: no education)  Primary | 1.15 | 0.90 - 1.47 | 0.270 |
| Secondary | 1.78 | 1.39 - 2.29 | <0.001 |
| Higher | 2.23 | 1.17 - 4.27 | 0.020 |
| **Wealth index** (ref: poorest)  Poorer | 2.06 | 1.53 - 2.77 | <0.001 |
| Middle | 3.11 | 2.22 - 4.34 | <0.001 |
| Richer | 4.20 | 2.90 - 6.08 | <0.001 |
| Richest | 11.08 | 7.03 - 17.47 | <0.001 |
| **Age at first birth** (continuous) | 1.12 | 1.07 - 1.16 | <0.001 |
| **Partner’s education** (ref: no education)  Primary | 1.06 | 0.79 - 1.41 | 0.700 |
| Secondary | 1.12 | 0.85 - 1.47 | 0.430 |
| Higher | 1.38 | 0.89 - 2.13 | 0.150 |
| **Partner’s age** (continuous) | 1.01 | 1.00 - 1.03 | 0.150 |

**Nepal 2016**

Table 26: Generalized Linear Models (GLMs) with binary outcome fitted to SBA access using sociodemographic factors.

| **Sociodemographic factors** | **OR** | **95% CI** | **p-value** |
| --- | --- | --- | --- |
| **Age** (continuous) | 0.92 | 0.89 - 0.94 | <0.001 |
| **Residence** – (ref: rural)  Urban | 1.33 | 1.01 - 1.75 | 0.050 |
| **Education** (ref: no education)  Primary | 1.41 | 1.10 - 1.82 | 0.010 |
| Secondary | 2.13 | 1.61 - 2.82 | <0.001 |
| Higher | 3.79 | 2.39 – 6.00 | <0.001 |
| **Wealth index** (ref: poorest)  Poorer | 1.66 | 1.23 - 2.23 | <0.001 |
| Middle | 3.29 | 2.43 - 4.45 | <0.001 |
| Richer | 4.06 | 2.94 - 5.60 | <0.001 |
| Richest | 8.08 | 4.63 - 14.1 | <0.001 |
| **Age at first birth** (continuous) | 1.13 | 1.10 - 1.17 | <0.001 |
| **Partner’s education** (ref: no education)  Primary | 1.10 | 0.84 - 1.45 | 0.490 |
| Secondary | 1.17 | 0.88 - 1.56 | 0.280 |
| Higher | 1.27 | 0.85 - 1.90 | 0.240 |
| **Partner’s age** (continuous) | 1.01 | 0.99 - 1.03 | 0.180 |

**Pakistan 1990**

Table 27: Generalized Linear Models (GLMs) with binary outcome fitted to SBA access using sociodemographic factors.

| **Sociodemographic factors** | **OR** | **95% CI** | **p-value** |
| --- | --- | --- | --- |
| **Age** (continuous) | 0.99 | 0.98 – 1.00 | 0.170 |
| **Residence** – (ref: rural)  Urban | 2.36 | 1.79 - 3.11 | <0.001 |
| **Education** (ref: no education)  Primary | 1.63 | 1.16 - 2.30 | 0.010 |
| Secondary | 2.57 | 1.80 - 3.66 | <0.001 |
| Higher | 22.27 | 3.35 - 147.81 | <0.001 |
| **Wealth index** (ref: poorest)  Poorer | 0.99 | 0.65 - 1.50 | 0.960 |
| Middle | 0.90 | 0.62 - 1.32 | 0.600 |
| Richer | 1.63 | 1.07 - 2.48 | 0.020 |
| Richest | 2.62 | 1.70 - 4.05 | <0.001 |
| **Age at first birth** (continuous) | 1.01 | 0.98 - 1.04 | 0.570 |
| **Partner’s education** (ref: no education)  Primary | 0.82 | 0.62 - 1.09 | 0.180 |
| Secondary | 1.20 | 0.93 - 1.56 | 0.170 |
| Higher | 1.38 | 0.89 - 2.13 | 0.150 |
| **Partner’s age** (continuous) | 1.31 | 0.81 - 2.12 | 0.270 |

**Pakistan 2006**

Table 28: Generalized Linear Models (GLMs) with binary outcome fitted to SBA access using sociodemographic factors.

| **Sociodemographic factors** | **OR** | **95% CI** | **p-value** |
| --- | --- | --- | --- |
| **Age** (continuous) | 0.95 | 0.94 - 0.97 | <0.001 |
| **Residence** – (ref: rural)  Urban | 1.50 | 1.24 - 1.82 | <0.001 |
| **Education** (ref: no education)  Primary | 1.41 | 1.18 - 1.67 | <0.001 |
| Secondary | 2.00 | 1.59 - 2.52 | <0.001 |
| Higher | 4.05 | 2.60 - 6.30 | <0.001 |
| **Wealth index** (ref: poorest)  Poorer | 1.34 | 1.04 - 1.72 | 0.020 |
| Middle | 1.88 | 1.43 - 2.48 | <0.001 |
| Richer | 2.70 | 2.04 - 3.59 | <0.001 |
| Richest | 4.61 | 3.25 - 6.53 | <0.001 |
| **Age at first birth** (continuous) | 1.06 | 1.04 - 1.08 | <0.001 |
| **Partner’s education** (ref: no education)  Primary | 1.10 | 0.88 - 1.37 | 0.390 |
| Secondary | 1.29 | 1.08 - 1.54 | 0.010 |
| Higher | 1.64 | 1.27 - 2.12 | <0.001 |
| **Partner’s age** (continuous) | 1.01 | 1.00 - 1.02 | 0.080 |

**Pakistan 2012**

Table 29: Generalized Linear Models (GLMs) with binary outcome fitted to SBA access using sociodemographic factors.

| **Sociodemographic factors** | **OR** | **95% CI** | **p-value** |
| --- | --- | --- | --- |
| **Age** (continuous) | 0.94 | 0.93 - 0.96 | <0.001 |
| **Residence** – (ref: rural)  Urban | 1.18 | 0.91 - 1.53 | 0.210 |
| **Education** (ref: no education)  Primary | 1.33 | 1.03 - 1.73 | 0.030 |
| Secondary | 1.87 | 1.48 - 2.36 | <0.001 |
| Higher | 3.72 | 2.42 - 5.71 | <0.001 |
| **Wealth index** (ref: poorest)  Poorer | 1.15 | 0.91 - 1.46 | 0.240 |
| Middle | 1.50 | 1.15 - 1.96 | <0.001 |
| Richer | 2.58 | 1.94 - 3.42 | <0.001 |
| Richest | 4.20 | 2.70 - 6.53 | <0.001 |
| **Age at first birth** (continuous) | 1.08 | 1.05 - 1.10 | <0.001 |
| **Partner’s education** (ref: no education)  Primary | 1.06 | 0.84 - 1.33 | 0.630 |
| Secondary | 1.33 | 1.10 - 1.60 | <0.001 |
| Higher | 1.75 | 1.36 - 2.27 | <0.001 |
| **Partner’s age** (continuous) | 1.01 | 1.00 - 1.03 | 0.090 |

**Pakistan 2017**

Table 30: Generalized Linear Models (GLMs) with binary outcome fitted to SBA access using sociodemographic factors.

| **Sociodemographic factors** | **OR** | **95% CI** | **p-value** |
| --- | --- | --- | --- |
| **Age** (continuous) | 0.95 | 0.93 - 0.97 | <0.001 |
| **Residence** – (ref: rural)  Urban | 1.17 | 0.87 - 1.58 | 0.310 |
| **Education** (ref: no education)  Primary | 1.32 | 1.02 - 1.7 | 0.030 |
| Secondary | 1.76 | 1.31 - 2.37 | <0.001 |
| Higher | 3.55 | 2.15 - 5.87 | <0.001 |
| **Wealth index** (ref: poorest)  Poorer | 1.16 | 0.89 - 1.52 | 0.270 |
| Middle | 1.89 | 1.39 - 2.58 | <0.001 |
| Richer | 2.84 | 1.85 - 4.36 | <0.001 |
| Richest | 4.15 | 2.47 - 6.97 | <0.001 |
| **Age at first birth** (continuous) | 1.06 | 1.03 - 1.09 | <0.001 |
| **Partner’s education** (ref: no education)  Primary | 0.90 | 0.70 - 1.16 | 0.430 |
| Secondary | 1.10 | 0.88 - 1.38 | 0.390 |
| Higher | 1.71 | 1.25 - 2.33 | <0.001 |
| **Partner’s age** (continuous) | 1.01 | 0.99 - 1.03 | 0.290 |

**Philippines 1993**

Table 31: Generalized Linear Models (GLMs) with binary outcome fitted to SBA access using sociodemographic factors.

| **Sociodemographic factors** | **OR** | **95% CI** | **p-value** |
| --- | --- | --- | --- |
| **Age** (continuous) | 0.82 | 0.81 - 0.83 | <0.001 |
| **Residence** – (ref: rural)  Urban | 1.14 | 1.00 - 1.29 | 0.050 |
| **Education** (ref: no education)  Primary | 2.13 | 1.32 - 3.43 | <0.001 |
| Secondary | 1.80 | 1.12 - 2.91 | 0.020 |
| Higher | 2.01 | 1.22 - 3.33 | 0.010 |
| **Wealth index** (ref: poorest)  Poorer | 0.92 | 0.77 - 1.11 | 0.400 |
| Middle | 0.80 | 0.66 - 0.97 | 0.030 |
| Richer | 0.58 | 0.48 - 0.72 | <0.001 |
| Richest | 0.48 | 0.38 - 0.60 | <0.001 |
| **Age at first birth** (continuous) | 1.13 | 1.11 - 1.14 | <0.001 |
| **Partner’s education** (ref: no education)  Primary | 2.36 | 1.36 - 4.09 | <0.001 |
| Secondary | 2.17 | 1.24 - 3.80 | 0.010 |
| Higher | 1.64 | 0.93 - 2.92 | 0.090 |
| **Partner’s age** (continuous) | 0.99 | 0.89 - 1.11 | 0.910 |

**Philippines 1998**

Table 32: Generalized Linear Models (GLMs) with binary outcome fitted to SBA access using sociodemographic factors.

| **Sociodemographic factors** | **OR** | **95% CI** | **p-value** |
| --- | --- | --- | --- |
| **Age** (continuous) | 0.97 | 0.95 - 0.99 | <0.001 |
| **Residence** – (ref: rural)  Urban | 2.59 | 2.04 - 3.27 | <0.001 |
| **Education** (ref: no education)  Primary | 1.79 | 0.95 - 3.38 | 0.070 |
| Secondary | 3.00 | 1.57 - 5.71 | <0.001 |
| Higher | 4.16 | 2.09 - 8.25 | <0.001 |
| **Wealth index** (ref: poorest)  Poorer | 1.76 | 1.42 - 2.18 | <0.001 |
| Middle | 3.41 | 2.64 - 4.4 | <0.001 |
| Richer | 4.75 | 3.41 - 6.62 | <0.001 |
| Richest | 7.71 | 4.87 - 12.2 | <0.001 |
| **Age at first birth** (continuous) | 1.08 | 1.05 - 1.10 | <0.001 |
| **Partner’s education** (ref: no education)  Primary | 4.61 | 1.79 - 11.88 | <0.001 |
| Secondary | 7.35 | 2.86 - 18.85 | <0.001 |
| Higher | 10.63 | 4.07 - 27.74 | <0.001 |
| **Partner’s age** (continuous) | 1.01 | 0.99 - 1.03 | 0.270 |

**Philippines 2003**

Table 33: Generalized Linear Models (GLMs) with binary outcome fitted to SBA access using sociodemographic factors.

| **Sociodemographic factors** | **OR** | **95% CI** | **p-value** |
| --- | --- | --- | --- |
| **Age** (continuous) | 0.97 | 0.95 - 0.98 | <0.001 |
| **Residence** – (ref: rural)  Urban | 2.55 | 2.00 - 3.25 | <0.001 |
| **Education** (ref: no education)  Primary | 2.82 | 1.36 - 5.82 | 0.010 |
| Secondary | 4.55 | 2.14 - 9.67 | <0.001 |
| Higher | 7.49 | 3.46 - 16.23 | <0.001 |
| **Wealth index** (ref: poorest)  Poorer | 1.80 | 1.46 - 2.22 | <0.001 |
| Middle | 2.94 | 2.26 - 3.82 | <0.001 |
| Richer | 4.65 | 3.35 - 6.45 | <0.001 |
| Richest | 7.05 | 4.42 - 11.24 | <0.001 |
| **Age at first birth** (continuous) | 1.07 | 1.05 - 1.10 | <0.001 |
| **Partner’s education** (ref: no education)  Primary | 2.23 | 1.15 - 4.33 | 0.020 |
| Secondary | 2.92 | 1.50 - 5.72 | <0.001 |
| Higher | 3.90 | 1.91 - 7.94 | <0.001 |
| **Partner’s age** (continuous) | 1.01 | 1.00 - 1.03 | 0.130 |

**Philippines 2008**

Table 34: Generalized Linear Models (GLMs) with binary outcome fitted to SBA access using sociodemographic factors.

| **Sociodemographic factors** | **OR** | **95% CI** | **p-value** |
| --- | --- | --- | --- |
| **Age** (continuous) | 0.98 | 0.96 – 1.00 | 0.060 |
| **Residence** – (ref: rural)  Urban | 1.64 | 1.30 - 2.08 | <0.001 |
| **Education** (ref: no education)  Primary | 1.29 | 0.66 - 2.54 | 0.460 |
| Secondary | 2.16 | 1.10 - 4.24 | 0.030 |
| Higher | 2.88 | 1.41 - 5.89 | <0.001 |
| **Wealth index** (ref: poorest)  Poorer | 2.49 | 2.01 - 3.09 | <0.001 |
| Middle | 4.56 | 3.42 - 6.07 | <0.001 |
| Richer | 6.02 | 4.35 - 8.33 | <0.001 |
| Richest | 17.92 | 10.02 - 32.04 | <0.001 |
| **Age at first birth** (continuous) | 1.07 | 1.04 - 1.09 | <0.001 |
| **Partner’s education** (ref: no education)  Primary | 6.04 | 2.17 - 16.85 | <0.001 |
| Secondary | 8.31 | 2.99 - 23.11 | <0.001 |
| Higher | 11.46 | 4.04 - 32.51 | <0.001 |
| **Partner’s age** (continuous) | 1.00 | 0.99 - 1.02 | 0.710 |

**Philippines 2013**

Table 35: Generalized Linear Models (GLMs) with binary outcome fitted to SBA access using sociodemographic factors.

| **Sociodemographic factors** | **OR** | **95% CI** | **p-value** |
| --- | --- | --- | --- |
| **Age** (continuous) | 0.97 | 0.95 - 0.99 | <0.001 |
| **Residence** – (ref: rural)  Urban | 1.17 | 0.92 - 1.48 | 0.190 |
| **Education** (ref: no education)  Primary | 3.63 | 1.88 - 7.02 | <0.001 |
| Secondary | 5.21 | 2.71 - 10.02 | <0.001 |
| Higher | 6.75 | 3.34 - 13.66 | <0.001 |
| **Wealth index** (ref: poorest)  Poorer | 2.35 | 1.91 - 2.90 | <0.001 |
| Middle | 4.14 | 3.20 - 5.35 | <0.001 |
| Richer | 8.96 | 5.88 - 13.65 | <0.001 |
| Richest | 15.75 | 8.28 - 29.99 | <0.001 |
| **Age at first birth** (continuous) | 1.05 | 1.03 - 1.08 | <0.001 |
| **Partner’s education** (ref: no education)  Primary | 1.50 | 0.97 - 2.31 | 0.070 |
| Secondary | 2.41 | 1.53 - 3.78 | <0.001 |
| Higher | 3.03 | 1.82 - 5.03 | <0.001 |
| **Partner’s age** (continuous) | 1.00 | 0.99 - 1.02 | 0.810 |

**Philippines 2017**

Table 36: Generalized Linear Models (GLMs) with binary outcome fitted to SBA access using sociodemographic factors.

| **Sociodemographic factors** | **OR** | **95% CI** | **p-value** |
| --- | --- | --- | --- |
| **Age** (continuous) | 0.96 | 0.93 - 0.98 | <0.001 |
| **Residence** – (ref: rural)  Urban | 1.83 | 1.21 - 2.77 | <0.001 |
| **Education** (ref: no education)  Primary | 2.24 | 1.14 - 4.42 | 0.020 |
| Secondary | 4.39 | 2.21 - 8.71 | <0.001 |
| Higher | 8.57 | 4.05 - 18.13 | <0.001 |
| **Wealth index** (ref: poorest)  Poorer | 1.67 | 1.19 - 2.35 | <0.001 |
| Middle | 2.91 | 2.03 - 4.17 | <0.001 |
| Richer | 4.75 | 2.34 - 9.62 | <0.001 |
| Richest | 7.91 | 2.75 - 22.74 | <0.001 |
| **Age at first birth** (continuous) | 1.07 | 1.03 - 1.10 | <0.001 |
| **Partner’s education** (ref: no education)  Primary | 2.12 | 1.29 - 3.51 | <0.001 |
| Secondary | 3.89 | 2.23 - 6.78 | <0.001 |
| Higher | 5.27 | 2.83 - 9.84 | <0.001 |
| **Partner’s age** (continuous) | 1.01 | 0.99 - 1.03 | 0.360 |

**Timor Leste 2009**

Table 37: Generalized Linear Models (GLMs) with binary outcome fitted to SBA access using sociodemographic factors.

| **Sociodemographic factors** | **OR** | **95% CI** | **p-value** |
| --- | --- | --- | --- |
| **Age** (continuous) | 0.97 | 0.96 - 0.99 | <0.001 |
| **Residence** – (ref: rural)  Urban | 2.07 | 1.67 - 2.56 | <0.001 |
| **Education** (ref: no education)  Primary | 1.36 | 1.09 - 1.68 | 0.010 |
| Secondary | 2.08 | 1.67 - 2.59 | <0.001 |
| Higher | 4.12 | 2.02 - 8.39 | <0.001 |
| **Wealth index** (ref: poorest)  Poorer | 1.20 | 0.93 - 1.54 | 0.160 |
| Middle | 1.67 | 1.30 - 2.16 | <0.001 |
| Richer | 2.69 | 2.07 - 3.49 | <0.001 |
| Richest | 6.32 | 4.70 - 8.51 | <0.001 |
| **Age at first birth** (continuous) | 1.04 | 1.02 - 1.06 | <0.001 |
| **Partner’s education** (ref: no education)  Primary | 1.22 | 0.99 - 1.51 | 0.060 |
| Secondary | 1.53 | 1.24 - 1.89 | <0.001 |
| Higher | 2.22 | 1.49 - 3.30 | <0.001 |
| **Partner’s age** (continuous) | 1.00 | 0.99 - 1.02 | 0.400 |

**Timor Leste 2016**

Table 38: Generalized Linear Models (GLMs) with binary outcome fitted to SBA access using sociodemographic factors.

| **Sociodemographic factors** | **OR** | **95% CI** | **p-value** |
| --- | --- | --- | --- |
| **Age** (continuous) | 0.97 | 0.95 - 0.99 | 0.010 |
| **Residence** – (ref: rural)  Urban | 2.79 | 2.12 - 3.66 | <0.001 |
| **Education** (ref: no education)  Primary | 1.19 | 0.95 - 1.50 | 0.140 |
| Secondary | 1.61 | 1.26 - 2.06 | <0.001 |
| Higher | 3.47 | 1.93 - 6.25 | <0.001 |
| **Wealth index** (ref: poorest)  Poorer | 1.55 | 1.23 - 1.96 | <0.001 |
| Middle | 2.46 | 1.86 - 3.26 | <0.001 |
| Richer | 3.65 | 2.78 - 4.79 | <0.001 |
| Richest | 5.33 | 3.48 - 8.17 | <0.001 |
| **Age at first birth** (continuous) | 1.05 | 1.02 - 1.07 | <0.001 |
| **Partner’s education** (ref: no education)  Primary | 1.16 | 0.93 - 1.44 | 0.180 |
| Secondary | 1.66 | 1.34 - 2.05 | <0.001 |
| Higher | 2.68 | 1.74 - 4.13 | <0.001 |
| **Partner’s age** (continuous) | 1.00 | 0.99 - 1.02 | 0.800 |
