# Supplementary material for: Women’s education and coverage of skilled birth attendance: An assessment of Sustainable Development Goal 3.1 in the South and Southeast Asian Region
Source: PLoS One. 2020 Apr 21;15(4):e0231489. doi: 10.1371/journal.pone.0231489 (PMC7173780; doi:10.1371/journal.pone.0231489)
Supplement: S2 File — (DOCX) [file pone.0231489.s003.docx]

**Women's education and coverage of skilled birth attendance: An assessment of Sustainable Development Goal 3.1 in the South and Southeast Asian Region**

Results of the variance inflation factor (VIF) for the selected factors to assess multicollinearity for models fitted to each of the 38 surveys are listed below (Tables 1 – 38).

Table 1: Variance inflation factors (VIF) for model fitted to DHS of Afghanistan 2015

| **Sociodemographic factors** | **VIF** |
| --- | --- |
| Age | 3.183756 |
| Residence: Urban | 1.847856 |
| Education: Primary | 1.059089 |
| Education: Secondary | 1.125771 |
| Education: Higher | 1.104207 |
| Wealth index: Poorer | 1.713925 |
| Wealth index: Middle | 1.712635 |
| Wealth index: Rich | 1.819817 |
| Wealth index: Richest | 2.500914 |
| Age at first birth | 1.158799 |
| Partner’s education: Primary | 1.106503 |
| Partner’s education: Secondary | 1.200822 |
| Partner’s education: Higher | 1.211656 |
| Partner’s age | 2.942980 |

Table 2: Variance inflation factors (VIF) for model fitted to DHS of Bangladesh 1993

| **Sociodemographic factors** | **VIF** |
| --- | --- |
| Age | 1.101730 |
| Residence: Urban | 1.206070 |
| Education: Primary | 1.339433 |
| Education: Secondary | 1.886710 |
| Education: Higher | 1.570294 |
| Wealth index: Poorer | 1.519776 |
| Wealth index: Middle | 1.581561 |
| Wealth index: Rich | 1.788328 |
| Wealth index: Richest | 2.407595 |
| Age at first birth | 1.155370 |
| Partner’s education: Primary | 1.269480 |
| Partner’s education: Secondary | 1.682095 |
| Partner’s education: Higher | 2.041786 |
| Partner’s age | 1.003330 |

Table 3: Variance inflation factors (VIF) for model fitted to DHS of Bangladesh 1996

| **Sociodemographic factors** | **VIF** |
| --- | --- |
| Age | 2.691325 |
| Residence: Urban | 1.295245 |
| Education: Primary | 1.342511 |
| Education: Secondary | 1.899672 |
| Education: Higher | 1.683288 |
| Wealth index: Poorer | 1.615843 |
| Wealth index: Middle | 1.645414 |
| Wealth index: Rich | 1.790240 |
| Wealth index: Richest | 2.405206 |
| Age at first birth | 1.225741 |
| Partner’s education: Primary | 1.285290 |
| Partner’s education: Secondary | 1.656978 |
| Partner’s education: Higher | 2.076098 |
| Partner’s age | 2.559407 |

Table 4: Variance inflation factors (VIF) for model fitted to DHS of Bangladesh 1999

| **Sociodemographic factors** | **VIF** |
| --- | --- |
| Age | 2.616455 |
| Residence: Urban | 1.289868 |
| Education: Primary | 1.429078 |
| Education: Secondary | 2.202346 |
| Education: Higher | 2.054515 |
| Wealth index: Poorer | 1.571029 |
| Wealth index: Middle | 1.690655 |
| Wealth index: Rich | 1.862098 |
| Wealth index: Richest | 2.832448 |
| Age at first birth | 1.333346 |
| Partner’s education: Primary | 1.322482 |
| Partner’s education: Secondary | 1.798312 |
| Partner’s education: Higher | 2.412582 |
| Partner’s age | 2.359075 |

Table 5: Variance inflation factors (VIF) for model fitted to DHS of Bangladesh 2004

| **Sociodemographic factors** | **VIF** |
| --- | --- |
| Age | 2.839337 |
| Residence: Urban | 1.271489 |
| Education: Primary | 1.540521 |
| Education: Secondary | 2.214750 |
| Education: Higher | 2.110997 |
| Wealth index: Poorer | 1.575374 |
| Wealth index: Middle | 1.655932 |
| Wealth index: Rich | 1.840945 |
| Wealth index: Richest | 2.634615 |
| Age at first birth | 1.360755 |
| Partner’s education: Primary | 1.410475 |
| Partner’s education: Secondary | 1.754672 |
| Partner’s education: Higher | 2.219294 |
| Partner’s age | 2.570775 |

Table 6: Variance inflation factors (VIF) for model fitted to DHS of Bangladesh 2007

| **Sociodemographic factors** | **VIF** |
| --- | --- |
| Age | 2.946129 |
| Residence: Urban | 1.323663 |
| Education: Primary | 1.729061 |
| Education: Secondary | 2.523125 |
| Education: Higher | 2.392209 |
| Wealth index: Poorer | 1.677404 |
| Wealth index: Middle | 1.713409 |
| Wealth index: Rich | 1.953361 |
| Wealth index: Richest | 2.839478 |
| Age at first birth | 1.417819 |
| Partner’s education: Primary | 1.519959 |
| Partner’s education: Secondary | 1.920964 |
| Partner’s education: Higher | 2.375066 |
| Partner’s age | 2.580909 |

Table 7: Variance inflation factors (VIF) for model fitted to DHS of Bangladesh 2011

| **Sociodemographic factors** | **VIF** |
| --- | --- |
| Age | 2.903451 |
| Residence: Urban | 1.301692 |
| Education: Primary | 2.128588 |
| Education: Secondary | 3.030675 |
| Education: Higher | 2.498213 |
| Wealth index: Poorer | 1.624733 |
| Wealth index: Middle | 1.775711 |
| Wealth index: Rich | 1.998958 |
| Wealth index: Richest | 2.697966 |
| Age at first birth | 1.484590 |
| Partner’s education: Primary | 1.696137 |
| Partner’s education: Secondary | 2.135620 |
| Partner’s education: Higher | 2.437546 |
| Partner’s age | 2.533820 |

Table 8: Variance inflation factors (VIF) for model fitted to DHS of Bangladesh 2014

| **Sociodemographic factors** | **VIF** |
| --- | --- |
| Age | 2.531995 |
| Residence: Urban | 1.281066 |
| Education: Primary | 2.483688 |
| Education: Secondary | 3.438116 |
| Education: Higher | 2.863048 |
| Wealth index: Poorer | 1.612614 |
| Wealth index: Middle | 1.750106 |
| Wealth index: Rich | 2.005630 |
| Wealth index: Richest | 2.584411 |
| Age at first birth | 1.468500 |
| Partner’s education: Primary | 1.826386 |
| Partner’s education: Secondary | 2.300294 |
| Partner’s education: Higher | 2.545830 |
| Partner’s age | 2.215642 |

Table 9: Variance inflation factors (VIF) for model fitted to DHS of Cambodia 2000

| **Sociodemographic factors** | **VIF** |
| --- | --- |
| Age | 3.337001 |
| Residence: Urban | 1.163148 |
| Education: Primary | 1.391486 |
| Education: Secondary | 1.677394 |
| Education: Higher | 1.040861 |
| Wealth index: Poorer | 1.413667 |
| Wealth index: Middle | 1.414224 |
| Wealth index: Rich | 1.442775 |
| Wealth index: Richest | 1.757353 |
| Age at first birth | 1.304309 |
| Partner’s education: Primary | 1.918592 |
| Partner’s education: Secondary | 2.309565 |
| Partner’s education: Higher | 1.140052 |
| Partner’s age | 2.811178 |

Table 10: Variance inflation factors (VIF) for model fitted to DHS of Cambodia 2005

| **Sociodemographic factors** | **VIF** |
| --- | --- |
| Age | 3.516604 |
| Residence: Urban | 1.181298 |
| Education: Primary | 1.601990 |
| Education: Secondary | 1.926919 |
| Education: Higher | 1.165719 |
| Wealth index: Poorer | 1.454792 |
| Wealth index: Middle | 1.459214 |
| Wealth index: Rich | 1.515088 |
| Wealth index: Richest | 2.128507 |
| Age at first birth | 1.317112 |
| Partner’s education: Primary | 2.204230 |
| Partner’s education: Secondary | 2.595843 |
| Partner’s education: Higher | 3.021054 |
| Partner’s age | 1.477014 |

Table 11: Variance inflation factors (VIF) for model fitted to DHS of Cambodia 2010

| **Sociodemographic factors** | **VIF** |
| --- | --- |
| Age | 2.982068 |
| Residence: Urban | 1.629453 |
| Education: Primary | 1.936314 |
| Education: Secondary | 2.448105 |
| Education: Higher | 1.446502 |
| Wealth index: Poorer | 1.487378 |
| Wealth index: Middle | 1.514719 |
| Wealth index: Rich | 1.747992 |
| Wealth index: Richest | 2.893109 |
| Age at first birth | 1.274625 |
| Partner’s education: Primary | 2.699769 |
| Partner’s education: Secondary | 3.204905 |
| Partner’s education: Higher | 1.915771 |
| Partner’s age | 2.609066 |

Table 12: Variance inflation factors (VIF) for model fitted to DHS of Cambodia 2014

| **Sociodemographic factors** | **VIF** |
| --- | --- |
| Age | 2.946054 |
| Residence: Urban | 1.693616 |
| Education: Primary | 2.670903 |
| Education: Secondary | 3.168367 |
| Education: Higher | 1.829096 |
| Wealth index: Poorer | 1.522784 |
| Wealth index: Middle | 1.534676 |
| Wealth index: Rich | 1.767664 |
| Wealth index: Richest | 2.941461 |
| Age at first birth | 1.364465 |
| Partner’s education: Primary | 3.452831 |
| Partner’s education: Secondary | 3.964766 |
| Partner’s education: Higher | 2.512983 |
| Partner’s age | 2.441396 |

Table 13: Variance inflation factors (VIF) for model fitted to DHS of India 1992

| **Sociodemographic factors** | **VIF** |
| --- | --- |
| Age | 1.227541 |
| Residence: Urban | 1.557667 |
| Education: Primary | 1.239585 |
| Education: Secondary | 1.847150 |
| Education: Higher | 1.753288 |
| Wealth index: Poorer | 1.648741 |
| Wealth index: Middle | 1.761736 |
| Wealth index: Rich | 2.158465 |
| Wealth index: Richest | 3.383206 |
| Age at first birth | 1.459164 |
| Partner’s education: Primary | 1.451717 |
| Partner’s education: Secondary | 1.892358 |
| Partner’s education: Higher | 1.895758 |
| Partner’s age | 1.000416 |

Table 14: Variance inflation factors (VIF) for model fitted to DHS of India 1998

| **Sociodemographic factors** | **VIF** |
| --- | --- |
| Age | 2.896839 |
| Residence: Urban | 1.444145 |
| Education: Primary | 1.233217 |
| Education: Secondary | 1.754274 |
| Education: Higher | 2.066307 |
| Wealth index: Poorer | 1.667480 |
| Wealth index: Middle | 1.836902 |
| Wealth index: Rich | 2.265917 |
| Wealth index: Richest | 3.174462 |
| Age at first birth | 1.617742 |
| Partner’s education: Primary | 1.442071 |
| Partner’s education: Secondary | 1.983887 |
| Partner’s education: Higher | 2.277959 |
| Partner’s age | 2.455200 |

Table 15: Variance inflation factors (VIF) for model fitted to DHS of India 2006

| **Sociodemographic factors** | **VIF** |
| --- | --- |
| Age | 3.180099 |
| Residence: Urban | 1.404620 |
| Education: Primary | 1.296357 |
| Education: Secondary | 2.103638 |
| Education: Higher | 2.284108 |
| Wealth index: Poorer | 1.779110 |
| Wealth index: Middle | 2.060593 |
| Wealth index: Rich | 2.652324 |
| Wealth index: Richest | 3.739075 |
| Age at first birth | 1.694231 |
| Partner’s education: Primary | 1.493760 |
| Partner’s education: Secondary | 2.313476 |
| Partner’s education: Higher | 2.468129 |
| Partner’s age | 2.663109 |

Table 16: Variance inflation factors (VIF) for model fitted to DHS of India 2015

| **Sociodemographic factors** | **VIF** |
| --- | --- |
| Age | 3.534748 |
| Residence: Urban | 1.294537 |
| Education: Primary | 1.413828 |
| Education: Secondary | 2.249108 |
| Education: Higher | 2.265831 |
| Wealth index: Poorer | 1.662065 |
| Wealth index: Middle | 1.840715 |
| Wealth index: Rich | 2.074646 |
| Wealth index: Richest | 2.566068 |
| Age at first birth | 1.595178 |
| Partner’s education: Primary | 1.695300 |
| Partner’s education: Secondary | 2.662731 |
| Partner’s education: Higher | 2.568670 |
| Partner’s age | 2.831744 |

Table 17: Variance inflation factors (VIF) for model fitted to DHS of Indonesia 1997

| **Sociodemographic factors** | **VIF** |
| --- | --- |
| Age | 1.209803 |
| Residence: Urban | 1.480199 |
| Education: Primary | 3.749006 |
| Education: Secondary | 4.566423 |
| Education: Higher | 2.155521 |
| Wealth index: Poorer | 1.376976 |
| Wealth index: Middle | 1.447613 |
| Wealth index: Rich | 1.646765 |
| Wealth index: Richest | 2.213873 |
| Age at first birth | 1.305257 |
| Partner’s education: Primary | 5.406945 |
| Partner’s education: Secondary | 6.188069 |
| Partner’s education: Higher | 2.892836 |
| Partner’s age | 1.001484 |

Table 18: Variance inflation factors (VIF) for model fitted to DHS of Indonesia 2002

| **Sociodemographic factors** | **VIF** |
| --- | --- |
| Age | 2.944544 |
| Residence: Urban | 1.531349 |
| Education: Primary | 7.154915 |
| Education: Secondary | 8.179047 |
| Education: Higher | 3.557378 |
| Wealth index: Poorer | 1.376798 |
| Wealth index: Middle | 1.521254 |
| Wealth index: Rich | 1.748937 |
| Wealth index: Richest | 2.317895 |
| Age at first birth | 1.440180 |
| Partner’s education: Primary | 10.143336 |
| Partner’s education: Secondary | 11.255406 |
| Partner’s education: Higher | 4.980230 |
| Partner’s age | 2.664087 |

Table 19: Variance inflation factors (VIF) for model fitted to DHS of Indonesia 2007

| **Sociodemographic factors** | **VIF** |
| --- | --- |
| Age | 2.957605 |
| Residence: Urban | 1.486161 |
| Education: Primary | 7.959063 |
| Education: Secondary | 9.226411 |
| Education: Higher | 4.160971 |
| Wealth index: Poorer | 1.432521 |
| Wealth index: Middle | 1.540415 |
| Wealth index: Rich | 1.791379 |
| Wealth index: Richest | 2.246541 |
| Age at first birth | 1.457354 |
| Partner’s education: Primary | 9.438907 |
| Partner’s education: Secondary | 10.875068 |
| Partner’s education: Higher | 4.996555 |
| Partner’s age | 2.619737 |

Table 20: Variance inflation factors (VIF) for model fitted to DHS of Indonesia 2012

| **Sociodemographic factors** | **VIF** |
| --- | --- |
| Age | 2.993833 |
| Residence: Urban | 1.312315 |
| Education: Primary | 10.069698 |
| Education: Secondary | 12.307664 |
| Education: Higher | 7.065944 |
| Wealth index: Poorer | 1.473374 |
| Wealth index: Middle | 1.590174 |
| Wealth index: Rich | 1.728786 |
| Wealth index: Richest | 2.021237 |
| Age at first birth | 1.463508 |
| Partner’s education: Primary | 13.362169 |
| Partner’s education: Secondary | 16.023353 |
| Partner’s education: Higher | 8.424973 |
| Partner’s age | 2.654599 |

Table 21: Variance inflation factors (VIF) for model fitted to DHS of Myanmar 2016

| **Sociodemographic factors** | **VIF** |
| --- | --- |
| Age | 2.982730 |
| Residence: Urban | 1.489286 |
| Education: Primary | 2.452472 |
| Education: Secondary | 2.918095 |
| Education: Higher | 2.296728 |
| Wealth index: Poorer | 1.454046 |
| Wealth index: Middle | 1.511634 |
| Wealth index: Rich | 1.714639 |
| Wealth index: Richest | 2.283974 |
| Age at first birth | 1.479591 |
| Partner’s education: Primary | 2.394056 |
| Partner’s education: Secondary | 2.822025 |
| Partner’s education: Higher | 1.983813 |
| Partner’s age | 2.500230 |

Table 22: Variance inflation factors (VIF) for model fitted to DHS of Nepal 1996

| **Sociodemographic factors** | **VIF** |
| --- | --- |
| Age | 3.293189 |
| Residence: Urban | 1.307682 |
| Education: Primary | 1.134461 |
| Education: Secondary | 1.336283 |
| Education: Higher | 1.223708 |
| Wealth index: Poorer | 1.448138 |
| Wealth index: Middle | 1.472185 |
| Wealth index: Rich | 1.493799 |
| Wealth index: Richest | 1.897525 |
| Age at first birth | 1.245238 |
| Partner’s education: Primary | 1.287509 |
| Partner’s education: Secondary | 1.597640 |
| Partner’s education: Higher | 1.367315 |
| Partner’s age | 2.920513 |

Table 23: Variance inflation factors (VIF) for model fitted to DHS of Nepal 2001

| **Sociodemographic factors** | **VIF** |
| --- | --- |
| Age | 3.847335 |
| Residence: Urban | 1.342584 |
| Education: Primary | 1.151628 |
| Education: Secondary | 1.543373 |
| Education: Higher | 1.245843 |
| Wealth index: Poorer | 1.442644 |
| Wealth index: Middle | 1.431439 |
| Wealth index: Rich | 1.469374 |
| Wealth index: Richest | 2.091778 |
| Age at first birth | 1.212817 |
| Partner’s education: Primary | 1.360424 |
| Partner’s education: Secondary | 1.711672 |
| Partner’s education: Higher | 1.567040 |
| Partner’s age | 3.483484 |

Table 24: Variance inflation factors (VIF) for model fitted to DHS of Nepal 2006

| **Sociodemographic factors** | **VIF** |
| --- | --- |
| Age | 3.735944 |
| Residence: Urban | 1.294760 |
| Education: Primary | 1.226395 |
| Education: Secondary | 1.726157 |
| Education: Higher | 1.532820 |
| Wealth index: Poorer | 1.437115 |
| Wealth index: Middle | 1.417254 |
| Wealth index: Rich | 1.562330 |
| Wealth index: Richest | 2.140490 |
| Age at first birth | 1.335609 |
| Partner’s education: Primary | 1.702786 |
| Partner’s education: Secondary | 2.177266 |
| Partner’s education: Higher | 2.024050 |
| Partner’s age | 3.188733 |

Table 25: Variance inflation factors (VIF) for model fitted to DHS of Nepal 2011

| **Sociodemographic factors** | **VIF** |
| --- | --- |
| Age | 3.374139 |
| Residence: Urban | 1.329034 |
| Education: Primary | 1.354793 |
| Education: Secondary | 2.001512 |
| Education: Higher | 1.940022 |
| Wealth index: Poorer | 1.425327 |
| Wealth index: Middle | 1.492376 |
| Wealth index: Rich | 1.740351 |
| Wealth index: Richest | 2.364595 |
| Age at first birth | 1.377483 |
| Partner’s education: Primary | 1.906398 |
| Partner’s education: Secondary | 2.659652 |
| Partner’s education: Higher | 2.432673 |
| Partner’s age | 2.944022 |

Table 26: Variance inflation factors (VIF) for model fitted to DHS of Nepal 2016

| **Sociodemographic factors** | **VIF** |
| --- | --- |
| Age | 3.460277 |
| Residence: Urban | 1.123714 |
| Education: Primary | 1.460518 |
| Education: Secondary | 2.068003 |
| Education: Higher | 2.248691 |
| Wealth index: Poorer | 1.516011 |
| Wealth index: Middle | 1.503707 |
| Wealth index: Rich | 1.565731 |
| Wealth index: Richest | 1.778066 |
| Age at first birth | 1.527876 |
| Partner’s education: Primary | 2.231908 |
| Partner’s education: Secondary | 3.094507 |
| Partner’s education: Higher | 2.976281 |
| Partner’s age | 2.774859 |

Table 27: Variance inflation factors (VIF) for model fitted to DHS of Pakistan 1990

| **Sociodemographic factors** | **VIF** |
| --- | --- |
| Age | 1.258472 |
| Residence: Urban | 1.674615 |
| Education: Primary | 1.137951 |
| Education: Secondary | 1.539264 |
| Education: Higher | 1.279845 |
| Wealth index: Poorer | 1.883141 |
| Wealth index: Middle | 2.214329 |
| Wealth index: Rich | 2.888286 |
| Wealth index: Richest | 3.936240 |
| Age at first birth | 1.278569 |
| Partner’s education: Primary | 1.170032 |
| Partner’s education: Secondary | 1.528997 |
| Partner’s education: Higher | 1.520210 |
| Partner’s age | 1.005062 |

Table 28: Variance inflation factors (VIF) for model fitted to DHS of Pakistan 2006

| **Sociodemographic factors** | **VIF** |
| --- | --- |
| Age | 2.672180 |
| Residence: Urban | 1.428323 |
| Education: Primary | 1.180898 |
| Education: Secondary | 1.445732 |
| Education: Higher | 1.587444 |
| Wealth index: Poorer | 1.597674 |
| Wealth index: Middle | 1.722922 |
| Wealth index: Rich | 2.036807 |
| Wealth index: Richest | 2.803965 |
| Age at first birth | 1.283395 |
| Partner’s education: Primary | 1.267021 |
| Partner’s education: Secondary | 1.586680 |
| Partner’s education: Higher | 1.714134 |
| Partner’s age | 2.355522 |

Table 29: Variance inflation factors (VIF) for model fitted to DHS of Pakistan 2012

| **Sociodemographic factors** | **VIF** |
| --- | --- |
| Age | 2.940986 |
| Residence: Urban | 1.437050 |
| Education: Primary | 1.234862 |
| Education: Secondary | 1.572778 |
| Education: Higher | 1.909919 |
| Wealth index: Poorer | 1.621245 |
| Wealth index: Middle | 1.808763 |
| Wealth index: Rich | 2.176044 |
| Wealth index: Richest | 3.017600 |
| Age at first birth | 1.367891 |
| Partner’s education: Primary | 1.307103 |
| Partner’s education: Secondary | 1.724224 |
| Partner’s education: Higher | 2.076909 |
| Partner’s age | 2.564909 |

Table 30: Variance inflation factors (VIF) for model fitted to DHS of Pakistan 2017

| **Sociodemographic factors** | **VIF** |
| --- | --- |
| Age | 2.957074 |
| Residence: Urban | 1.350448 |
| Education: Primary | 1.221601 |
| Education: Secondary | 1.554514 |
| Education: Higher | 1.946550 |
| Wealth index: Poorer | 1.680876 |
| Wealth index: Middle | 1.896735 |
| Wealth index: Rich | 2.225075 |
| Wealth index: Richest | 2.799950 |
| Age at first birth | 1.491033 |
| Partner’s education: Primary | 1.369047 |
| Partner’s education: Secondary | 1.852600 |
| Partner’s education: Higher | 2.117625 |
| Partner’s age | 2.496752 |

Table 31: Variance inflation factors (VIF) for model fitted to DHS of Philippines 1993

| **Sociodemographic factors** | **VIF** |
| --- | --- |
| Age | 1.200938 |
| Residence: Urban | 1.207833 |
| Education: Primary | 10.405783 |
| Education: Secondary | 10.215344 |
| Education: Higher | 8.998163 |
| Wealth index: Poorer | 1.653531 |
| Wealth index: Middle | 1.808423 |
| Wealth index: Rich | 1.998986 |
| Wealth index: Richest | 2.376355 |
| Age at first birth | 1.242748 |
| Partner’s education: Primary | 12.618726 |
| Partner’s education: Secondary | 12.241849 |
| Partner’s education: Higher | 10.356288 |
| Partner’s age | 1.000993 |

Table 32: Variance inflation factors (VIF) for model fitted to DHS of Philippines 1998

| **Sociodemographic factors** | **VIF** |
| --- | --- |
| Age | 2.914051 |
| Residence: Urban | 1.303312 |
| Education: Primary | 10.607994 |
| Education: Secondary | 11.649726 |
| Education: Higher | 10.633949 |
| Wealth index: Poorer | 1.467426 |
| Wealth index: Middle | 1.645476 |
| Wealth index: Rich | 1.912914 |
| Wealth index: Richest | 2.033219 |
| Age at first birth | 1.458382 |
| Partner’s education: Primary | 13.230528 |
| Partner’s education: Secondary | 13.256294 |
| Partner’s education: Higher | 12.236896 |
| Partner’s age | 2.570699 |

Table 33: Variance inflation factors (VIF) for model fitted to DHS of Philippines 2003

| **Sociodemographic factors** | **VIF** |
| --- | --- |
| Age | 3.039253 |
| Residence: Urban | 1.307858 |
| Education: Primary | 12.332012 |
| Education: Secondary | 15.206323 |
| Education: Higher | 13.608509 |
| Wealth index: Poorer | 1.581935 |
| Wealth index: Middle | 1.791703 |
| Wealth index: Rich | 1.987422 |
| Wealth index: Richest | 2.277831 |
| Age at first birth | 1.482285 |
| Partner’s education: Primary | 11.031503 |
| Partner’s education: Secondary | 12.154374 |
| Partner’s education: Higher | 11.544776 |
| Partner’s age | 2.652240 |

Table 34: Variance inflation factors (VIF) for model fitted to DHS of Philippines 2008

| **Sociodemographic factors** | **VIF** |
| --- | --- |
| Age | 2.914020 |
| Residence: Urban | 1.266514 |
| Education: Primary | 11.876704 |
| Education: Secondary | 16.389690 |
| Education: Higher | 14.161085 |
| Wealth index: Poorer | 1.590488 |
| Wealth index: Middle | 1.726950 |
| Wealth index: Rich | 1.958654 |
| Wealth index: Richest | 2.142752 |
| Age at first birth | 1.531232 |
| Partner’s education: Primary | 12.506401 |
| Partner’s education: Secondary | 14.954819 |
| Partner’s education: Higher | 13.294628 |
| Partner’s age | 2.475386 |

Table 35: Variance inflation factors (VIF) for model fitted to DHS of Philippines 2013

| **Sociodemographic factors** | **VIF** |
| --- | --- |
| Age | 2.937537 |
| Residence: Urban | 1.214792 |
| Education: Primary | 10.808526 |
| Education: Secondary | 16.181745 |
| Education: Higher | 14.134256 |
| Wealth index: Poorer | 1.538801 |
| Wealth index: Middle | 1.707175 |
| Wealth index: Rich | 1.906834 |
| Wealth index: Richest | 2.104617 |
| Age at first birth | 1.482753 |
| Partner’s education: Primary | 11.737894 |
| Partner’s education: Secondary | 14.574215 |
| Partner’s education: Higher | 12.729339 |
| Partner’s age | 2.523988 |

Table 36: Variance inflation factors (VIF) for model fitted to DHS of Philippines 2017

| **Sociodemographic factors** | **VIF** |
| --- | --- |
| Age | 2.927488 |
| Residence: Urban | 1.164442 |
| Education: Primary | 12.693218 |
| Education: Secondary | 20.461609 |
| Education: Higher | 18.091162 |
| Wealth index: Poorer | 1.444475 |
| Wealth index: Middle | 1.578983 |
| Wealth index: Rich | 1.722699 |
| Wealth index: Richest | 1.830360 |
| Age at first birth | 1.551622 |
| Partner’s education: Primary | 12.988106 |
| Partner’s education: Secondary | 16.210313 |
| Partner’s education: Higher | 13.861228 |
| Partner’s age | 2.449090 |

Table 37: Variance inflation factors (VIF) for model fitted to DHS of Timor Leste 2009

| **Sociodemographic factors** | **VIF** |
| --- | --- |
| Age | 2.766747 |
| Residence: Urban | 1.262122 |
| Education: Primary | 1.506798 |
| Education: Secondary | 2.099046 |
| Education: Higher | 1.305981 |
| Wealth index: Poorer | 1.547189 |
| Wealth index: Middle | 1.608264 |
| Wealth index: Rich | 1.728062 |
| Wealth index: Richest | 2.159241 |
| Age at first birth | 1.318000 |
| Partner’s education: Primary | 1.612202 |
| Partner’s education: Secondary | 2.077399 |
| Partner’s education: Higher | 1.485423 |
| Partner’s age | 2.378746 |

Table 38: Variance inflation factors (VIF) for model fitted to DHS of Timor Leste 2016

| **Sociodemographic factors** | **VIF** |
| --- | --- |
| Age | 2.624606 |
| Residence: Urban | 1.490918 |
| Education: Primary | 1.591969 |
| Education: Secondary | 2.316214 |
| Education: Higher | 2.030099 |
| Wealth index: Poorer | 1.699255 |
| Wealth index: Middle | 1.800424 |
| Wealth index: Rich | 2.205742 |
| Wealth index: Richest | 2.716537 |
| Age at first birth | 1.332873 |
| Partner’s education: Primary | 1.629239 |
| Partner’s education: Secondary | 2.176536 |
| Partner’s education: Higher | 2.161010 |
| Partner’s age | 2.277002 |
