# Supplementary material for: Women’s education and coverage of skilled birth attendance: An assessment of Sustainable Development Goal 3.1 in the South and Southeast Asian Region
Source: PLoS One. 2020 Apr 21;15(4):e0231489. doi: 10.1371/journal.pone.0231489 (PMC7173780; doi:10.1371/journal.pone.0231489)
Supplement: Attachment — Submitted filename: General Comments.docx [file pone.0231489.s004.docx]

**General Comments**

1. This is a well written manuscript. The methods and statistical analysis is well executed.
2. Check the font size of line 176 on page 7.

**Supplementary material**

3. The Tables in the supplementary material are very informative and I wish the authors will consider referring them or presenting some of them in the result section.
4. Why is the intercept reported in the table? I don’t think this is adding anything to the result hence consider removing it.
5. There is no need repeating the reference category for variable category.
6. I think the tables in the supplementary material can be presented in a better way. See example of Table 1 below.
7. Consider reporting all p-values to 3 decimal place.

Table 1: Generalized Linear Models (GLMs) with binary outcome fitted to SBA access using sociodemographic factors.

| **Sociodemographic factors** | **OR** | **95% CI** | **P-value** |
| --- | --- | --- | --- |
| **Age** (continuous) | 1.00 | 0.98 - 1.01 | 0.52 |
| **Residence** |  |  |  |
| Rural (Reference) |  |  |  |
| Urban | 1.48 | 1.04 - 2.11 | 0.03 |
| **Education** |  |  |  |
| No education (Reference) |  |  |  |
| Primary | 2.23 | 1.73 – 2.88 | <0.001 |
| Secondary | 2.36 | 1.82 - 3.06 | <0.001 |
| Higher | 18.79 | 6.86 – 51.50 | <0.001 |
| **Wealth index** |  |  |  |
| Poorest (Reference) |  |  |  |
| Poorer | 1.89 | 1.63 – 2.19 | <0.001 |
| Middle | 2.45 | 1.87 – 3.20 | <0.001 |
| Richer | 5.06 | 3.98 – 6.42 | <0.001 |
| Richest | 9.66 | 6.33 – 14.75 | <0.001 |
| **Age at first birth** (continuous) | 1.07 | 1.04 – 1.09 | <0.001 |
| **Partner’s education** |  |  |  |
| No education (Reference) |  |  |  |
| Primary | 1.51 | 1.27 – 1.78 | <0.001 |
| Secondary | 1.79 | 1.51 – 2.13 | <0.001 |
| Higher | 2.04 | 1.56 – 2.66 | <0.001 |
| **Partner’s age** (continuous) | 0.99 | 0.98 – 1.00 | 0.04 |
